# A quantitative assessment of silicone and PTFE-based stamp techniques for restoring occlusal anatomy using resin-based composites

**DOI:** 10.1007/s00784-021-03992-8

**Published:** 2021-05-28

**Authors:** Christian Klein, Christiane von Ohle, Diana Wolff, Christian Meller

**Affiliations:** 1 Department of Conservative Dentistry, Periodontology and Endodontology, University Centre of Dentistry, Oral Medicine and Maxillofacial Surgery, University Hospital Tübingen, 72076 Tübingen, Germany; 2 Private practice Meller Zahngesundheit, Waiblingen, Germany

**Keywords:** Resin-based composite, Restoration, Stamp technique, Intraoral scanner, OraCheck, CEREC

## Abstract

**Objectives:**

Publications on stamp techniques for placing resin-based composite (RBC) restorations consist mainly of case studies. Furthermore, comparative studies are rare and no longer relevant to the materials tested today. Thus, two general techniques were investigated in this study.

**Materials and methods:**

Standardized occlusion class I cavities were prepared in twenty-eight extracted caries-free wisdom teeth with unimpaired occlusal surfaces and restored with the RBC material *Grandio*^*®*^. Light curing of the final layer was performed either after removal of the stamp isolated with PTFE tape or by leaving a stamp made of transparent polysiloxane in place. *CEREC* scans of the RBC restorations placed (follow-up) were superimposed on scans of the unimpaired occlusal surface (baseline) and quantitatively analyzed with the software *OraCheck* with regard to volume change and gain or loss of layer thickness in six sectional planes.

**Results:**

Assessing the excess material, there was no difference (p = 0.31) between the silicone technique (0.26 mm ± 0.02) and the PTFE technique (0.22 mm ± 0.02 mm). Nevertheless, the loss of tooth substance was significantly greater (p < 0.001) with the silicone technique (−0.29 mm ± 0.02 mm) than with the PTFE technique (−0.15 mm ± 0.02 mm).

**Conclusions:**

With the PTFE stamp technique, less healthy tooth structure was removed during the finishing procedure and the stamp was more dimensionally stable.

**Clinical relevance:**

The study shows the advantages and disadvantages of the investigated stamp techniques and helps the practitioner to choose an appropriate technique.

## Introduction

The development of stamp techniques largely coincides, in historical terms, with the introduction of resin-based composites (RBC) in dentistry. One reason for this is that previously applied restorative materials, like for instance amalgam, did not allow the use of a stamp technique. On the other hand, the operative procedures of RBC restorations are often considerably more time-consuming than that of amalgam restorations [1]. For this reason, methods were explored to facilitate the placing and finishing of RBC restorations. As early as 1968, N.O. Feeley published a method for the placement of class I restorations with the cold-curing RBC *Addent 12* (3M Dental Products, St. Paul, USA) using a plastic index and tin foil for isolation [2].

Over the past 50 years, two general stamp techniques have been established for the use of restorative light-curing RBCs, namely those using a transparent material for making the index/stamp. As the material is transparent, the stamp remains in its position on the tooth during the light curing of the RBC final surface layer [3]. Various transparent materials have been proposed for performing this stamp technique: polysiloxane bite registration materials [4–8], light-curable materials [9, 10], and commercially available occlusal transfer devices [11–13]. In contrast, the other proposed stamp technique removes the stamp before light curing the RBC. In this case, the material used for making the stamp does not necessarily need to be transparent. However, the stamp must be adequately isolated so that the unpolymerized RBC surface layer does not adhere to the stamp and thus becomes deformed when the index is removed. In addition to the tin foil already described above [2, 14], cling film [15], dentin adhesive [16], and more recently, PTFE tape [17, 18] have been proposed for isolation.

The majority of publications on indices/stamp techniques are case reports that describe the different variants of the abovementioned two general techniques. In contrast, evaluative research studies of the techniques are rare. In 1985, Rukmo et al. investigated two freehand and two stamp techniques (one ready-to-use and one individualized index) for contouring the occlusal surface of class II cavities [15]. It was shown that the freehand techniques caused less excess in comparison to the stamp techniques and, as a consequence, less damage to the natural tooth substance when removing the excess RBC material. Nevertheless, none of the contouring techniques tested was considered ideal. In 1998, Hamilton et al. were able to show that the time required to place and finish the restoration was reduced by the use of a clear occlusal index compared to applying a manual technique, but that there was no overall benefit when taking into account the time required to make the index [6]. The only advantage of the stamp technique was a significantly better surface smoothness. In 2013, Pitta Lopes et al. analyzed the influence of three occlusal index materials and their layer thickness on the light-curing effectiveness of an RBC [19]. Compared to two polysiloxanes (i.e., *Registrado Clear* and *Memosil-2*), the use of the occlusal transfer device *Bite-pref* (Bitepref Productos Dentales S. L., Malaga, Spain) resulted in a decrease in the polymerization rate at a depth of 2mm but prevented the formation of an oxygen inhibited layer better. An increase in the thickness of the index material from one to 2mm did not affect polymerization.

The aim of the present study was the quantitative assessment of the two described general stamp techniques for the rehabilitation of the occlusal surface of class I cavities by applying up-to-date materials and a modern evaluation technique (i.e., a digital, optical impression system and a software to digitally visualize and measure differences between virtual optical scans). The null hypothesis was that there are no differences between the two stamp techniques.

## Methodology

### Tooth selection and digitalization

Twenty-eight extracted caries-free wisdom teeth with unimpaired occlusal surfaces were collected in accordance with the ethical guidelines of the University Hospital Ethics Committee (approval protocol 694/2012BO2) and stored at room temperature in a 0.9 % saline solution during the entire experiment to prevent tooth dehydration. The teeth were mounted in the position of the 46 tooth in a lower jaw model (Frasaco GmbH, Tettnang, Germany) with polymethyl-methacrylate resin (*Technovit*^*®*^ *4004*, Kulzer GmbH, Hanau, Germany). In this way, the mounted tooth could be easily removed from the model to be scanned individually.

Surface scans of the teeth were performed using a *CEREC Omnicam* operated with the *CEREC SW 5.1.1* software (Dentsply Sirona Deutschland GmbH, Bensheim, Germany). The device was maintained according to the manufacturer’s instructions and the Directive 93/42/EEC (Medical Device Directive). The tooth was placed in the middle of a 15-cm diameter turntable (*SR01*, Cablematic Dos Mil SLU, Barcelona, Spain) rotating at a speed of 2.4 revolutions per minute (rpm). In the *acquisition phase*, the occlusal surface was scanned first at an angle of between 0 and 30° to the surface. When the digitalization of the occlusal surface was complete, the angle was changed to 90° (parallel to the surface of the table) to facilitate the scanning of the sides of the tooth. During the scanning process, the camera was positioned as close as possible to the tooth without interfering with the turntable rotation. The virtual tooth model was centered in the area of tooth 46 and the model axis was redefined, namely the *model phase*. All scans of the caries-free teeth with unimpaired occlusal surfaces were exported to the software *OraCheck 5.0.0* (Dentsply Sirona Deutschland GmbH, Bensheim, Germany) for superimposition and further analyses as the experimental protocol required.

### Cavity preparation

Standardized occlusal class I cavities were prepared using a water-cooled diamond bur by taking the form and dimensions of the bur (diameter and depth marking of the diamond-coated surface) as benchmark. The occlusal cavity of each tooth was prepared to a depth of 2 mm, a width of 3 mm, and a length of 6 mm, positioned at the central fissure, using a 5° tapered bur (845 KRD 02, Gebr. Brasseler GmbH & Co. KG, Lemgo, Germany). The prepared teeth were stored in 0.9 % sterile saline solution at room temperature unless moisture isolation was required for other aspects of the experimental protocol.

### RBC restoration

To each of the two groups (i.e., silicone stamp technique or PTFE stamp technique), 14 prepared teeth were assigned to be restored with the resin-based composite (RBC) *Grandio*^*®*^ (Shade A2, Lot: 1736598, VOCO GmbH, Cuxhaven, Germany). The restoration technique involved etching the entire cavity with *Ultra-Etch* (Lot: BHDFX, Ultradent Products GmbH, Cologne, Germany), and the RBC was bonded to the tooth structure with *OptiBond™ FL* (Prime Lot: 7038745, Adhesive Lot: 7101804, KerrHawe SA, Bioggio, Switzerland). RBC restorations were performed on all teeth using an oblique two-layering technique. The individual increments were light irradiated for 20 s with a *VALO*^*®*^ light-curing unit (Ultradent Products GmbH, Köln, Germany) with an output intensity of 1000 mW/cm^2^. The surface layers of the restorations were created by either the silicone stamp technique or the PTFE stamp technique.

### PTFE stamp technique

**Fig. 1:**
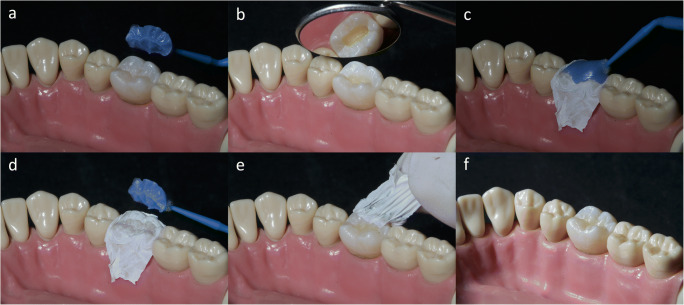
Rehabilitation of the occlusal surface with a resin-based composite (RCB) restoration using the PTFE stamp technique with a flowable light-cure resin paste (Paint-On Dental Dam, Lot: 1825300004, DenMat Holdings, USA) and PTFE tape (Unitape, Unipak A/S, Galten; Denmark): **a** light-cured stamp with a microbrush handle, **b** prepared and etched cavity, **c** forming the last layer, PTFE tape and stamp in position, **d** removed stamp with PTFE tape in situ, **e** removing the PTFE tape before excess removal and light curing, and **f** restored cavity (corresponds to *PTFEScan*)

The surface layers of the restoration were formed using a stamp made prior to cavity preparation (Fig. 1a–f) with a flowable light-cure resin paste (*Paint-On Dental Dam*, Lot: 1825300004, Den-Mat Holdings, Lompoc, USA) and an isolating 75-μm PTFE tape (*Unitape*^*®*^, Unipak A/S, Galten; Denmark). After removing the stamp and the PTFE tape, any excess of the RBC was carefully removed and only then was the restoration polymerized for 20 s by the light-curing lamp. The surface of the restoration was polished using the pre-polisher *Identoflex*^*TM*^ *Composite Polisher* (KerrHawe SA, Bioggio, Switzerland) and the high gloss polisher *Occlubrush*^*TM*^ (KerrHawe SA, Bioggio, Switzerland). Surface scans of the teeth with the restored class I cavities were performed using a *CEREC Omnicam* operated with the *CEREC SW 5.1.1* software in accordance with the scanning conditions outlined above (i.e., *PTFEScan*).

### Silicone stamp technique

**Fig. 2:**
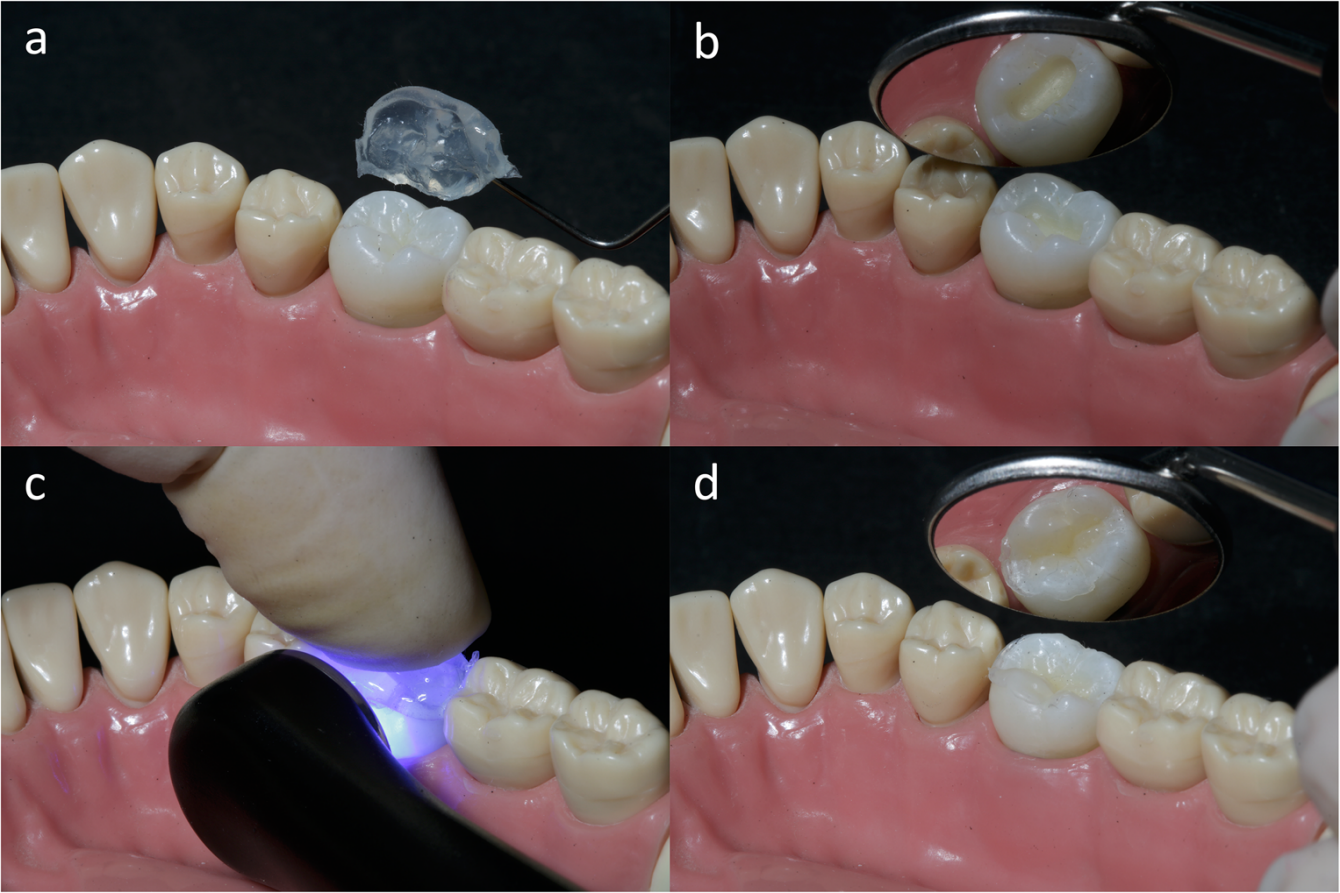
Rehabilitation of the occlusal surface with a resin-based composite (RCB) restoration using the silicone stamp technique with a transparent addition-vulcanizing 2-component vinyl polysiloxane (Regofix transparent, Lot: 911767.27, Dreve Dentamid GmbH, Unna, Germany): **a** silicone stamp, **b** prepared and etched cavity, **c** light-curing while a defined digital pressure of 5 N was applied, and **d** light-cured but unfinished restoration (corresponds to *SilScan1*)

The surface layers of the restoration were formed using a stamp made prior to cavity preparation (Fig. 2a–d) with a transparent addition-vulcanizing 2-component vinyl polysiloxane (*Regofix transparent*, Lot: 911767.27, Dreve Dentamid GmbH, Unna, Germany). The surface layer was polymerized for 20 s from the buccal and for 20 s from the lingual side of the stamp with the light-curing lamp while a defined digital pressure of 5 N was applied to the top of the silicone stamp. The pressure was controlled by placing the tooth on a balance (*Voltcraft TS-5000/1*, Conrad Electronic, Hirschau, Germany). The restoration was then light-cured from above for 20 s before and after the silicone stamp was removed. Surface scans of the teeth with the light-cured but unfinished restoration were performed using a *CEREC Omnicam* operated with the *CEREC SW 5.1.1* software in accordance with the scanning conditions outlined above (i.e., *SilScan1*).

Afterwards, any excess of the RBC was removed with a diamond bur (836.314.010, Gebr. Brasseler GmbH & Co. KG, Lemgo, Germany). The surfaces of the restoration were polished by the same procedure already described above using *Identoflex*^*TM*^ *Composite Polisher* and *Occlubrush*^*TM*^. Surface scans of the teeth with the restored class I cavities were performed using a *CEREC Omnicam* operated with the *CEREC SW 5.1.1* software in accordance with the scanning conditions outlined above (i.e., *SilScan2*).

All restorative procedures were done by a senior physician with more than 20 years of professional experience using a head-worn loupe at ×4.3 magnification (*EyeMag Pro S*, Carl Zeiss Vision GmbH, Aalen, Germany).

All scans of the respective processing stage (*PTFEScan*, *SilScan1*, and *SilScan2*) of the restored teeth were exported to the software *OraCheck 5.0.0* for superimposition and further analyses as the experimental protocol required.

### Data analysis

**Fig. 3:**
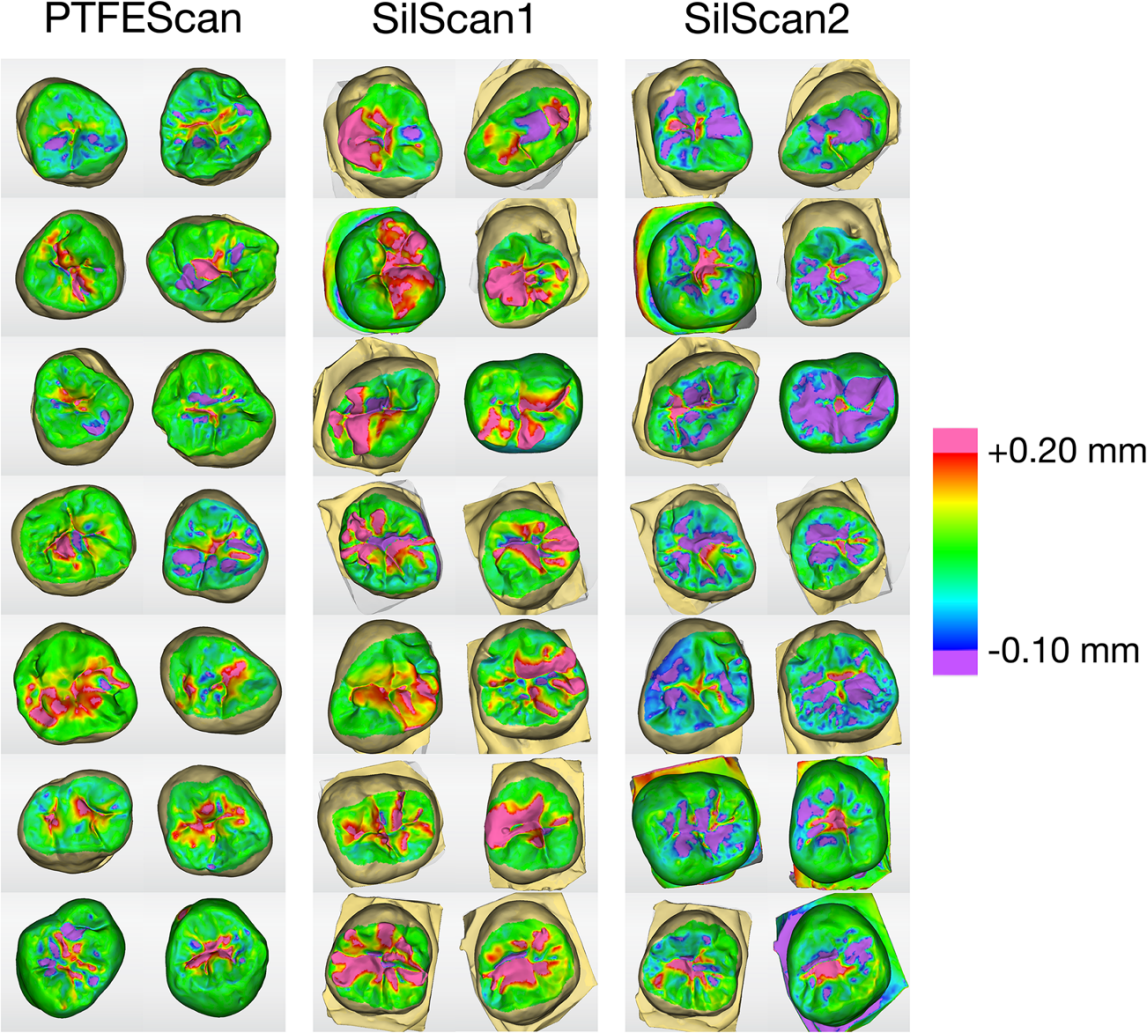
Visualization of the volume analysis between the respective processing stage (follow-up) and the unimpaired occlusal surface (baseline). Yellow, red, and pink shades show an increase and blue and violet shades show a decrease compared to the baseline. In the case of the finished restorations, excess is mainly located in the area of the fissures, while deficits are found on the cusp slopes. The occurrence of deficits clearly predominates in the *SilScan2* group in comparison to the *PTFEScan* group. In the *SilScan1* group, the areas with excess are predominant. Nevertheless, there are also teeth with a deficit in the fissure area. This indicates that the flexible silicone stamp has displaced composite in the area of the cavity below the level of the unimpaired surface

**Fig. 4:**
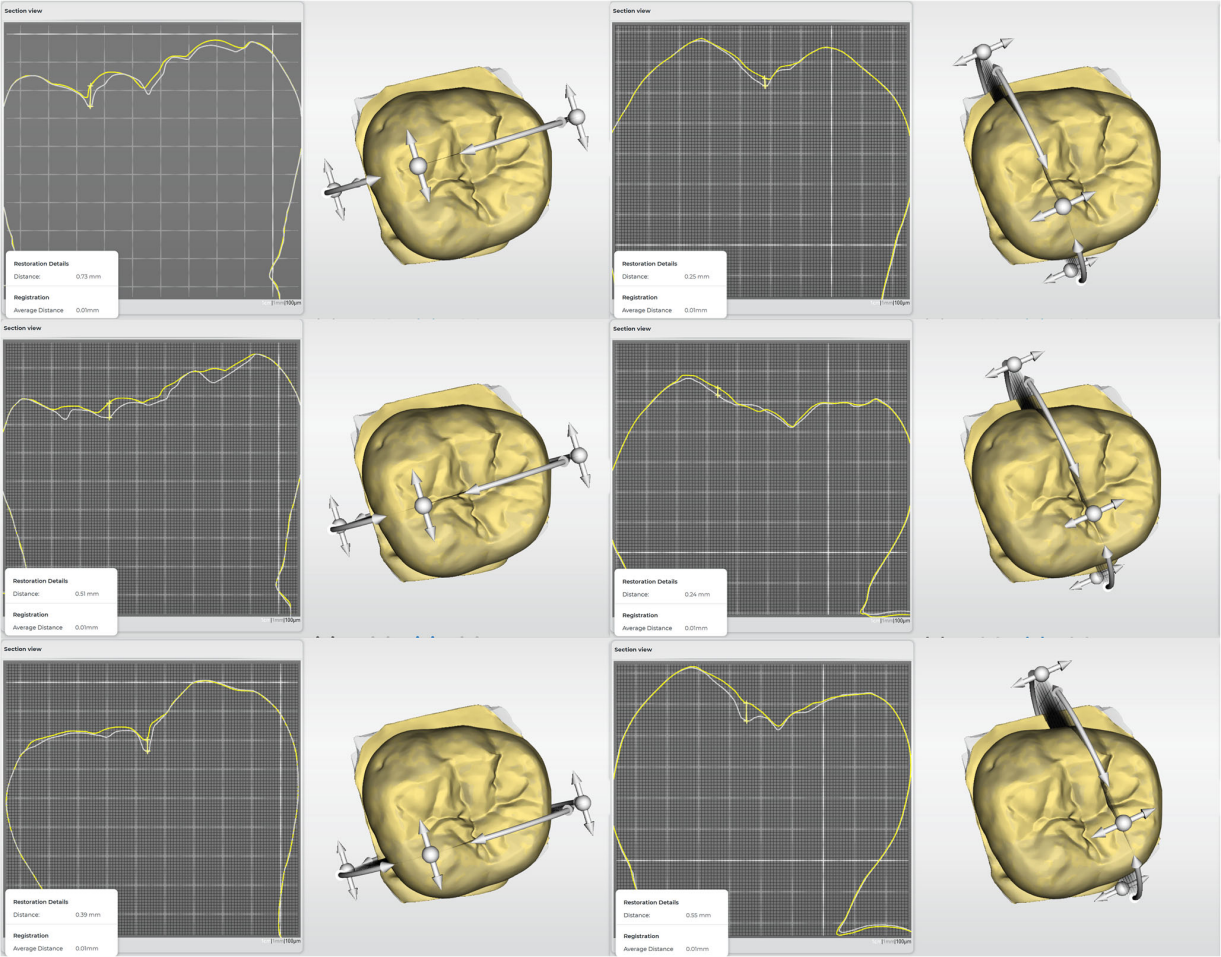
The screen shots illustrate the analysis of the maximum gain and loss for the six investigated sectional planes of each processing stage. These were placed in mesio-distal (left column) and oro-buccal (right column) alignment in the first, second, and third quarter of the distance between the marginal ridges and cusp tips, respectively. In each sectional plane, the point with the maximum gain and loss was measured perpendicular to the occlusal plane

For the superimposition of the scans, the unchanged sides of the scanned teeth were marked as arrangement region with the region tool. The percentage of the arrangement region, whose overlap distance after superimposition was less than 0.1 mm, was documented. The volume change was calculated by superimposing the scan of the respective processing stage (*PTFEScan*, *SilScan1*, and *SilScan2*) of the RBC restored tooth with the scan of the tooth with unimpaired occlusal surface as baseline using the software volume tool after marking the occlusal surface with the region tool (Fig. 3). The analysis of the maximum gain and loss in six sectional planes was investigated in the abovementioned superimpositions of each processing stage. The section planes were located in mesio-distal and oro-buccal alignment in the first, second, and third quarter of the distance between the crests of the marginal ridges (Fig. 4). In each sectional plane, the point with the maximum gain and loss was measured perpendicular to the occlusal plane. The investigator was blinded with regard to the stamp technique employed.

### Statistical analyses

Statistical analyses were carried out with the software *JMP 14.2* (SAS Institute Inc., Cary, USA). Firstly, the data (mean ± standard error of the mean) was checked for conformity with normal distribution by the Shapiro-Wilk W-test at p > 0.05. To assess statistically significant differences between the test data, the Dunn All Pairs for Joint Ranks test was performed at a significance level of α = 0.05.

## Results

The overlap distance 0.0–0.1 mm during the register process was (97 ± 2) % for *SilScan1*, (98 ± 2) % for *SilScan2*, and (99.4 ± 0.2) % for *PTFEScan* of the marked surface. The differences between the groups were not significant.

**Fig. 5:**
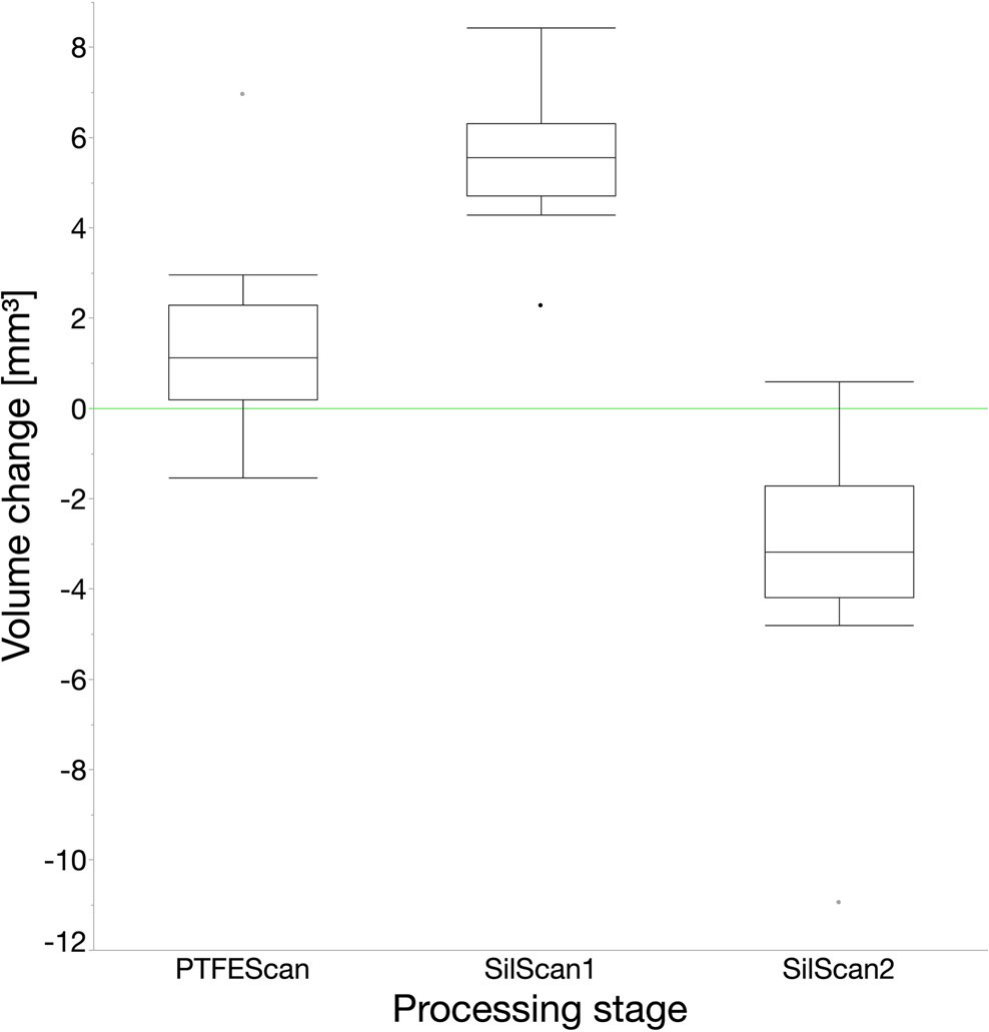
Boxplot showing the volume change of each processing stage and stamp technique in comparison to the unimpaired occlusal surface

The volume change in comparison to the unimpaired occlusal surface (Fig. 3 and Fig. 5) was (5.6 ± 0.4) mm^3^ for *SilScan1*, (1.3 ± 0.6) mm^3^ for *PTFEScan*, and (−3.3 ± 0.7) mm^3^ for *SilScan2*. The difference between *SilScan1* and *SilScan2* with p < 0.0001 and *PTFEScan* with p = 0.02 was significant. The difference between *SilScan2* and *PTFEScan* was also significant (p = 0.01).

**Fig. 6:**
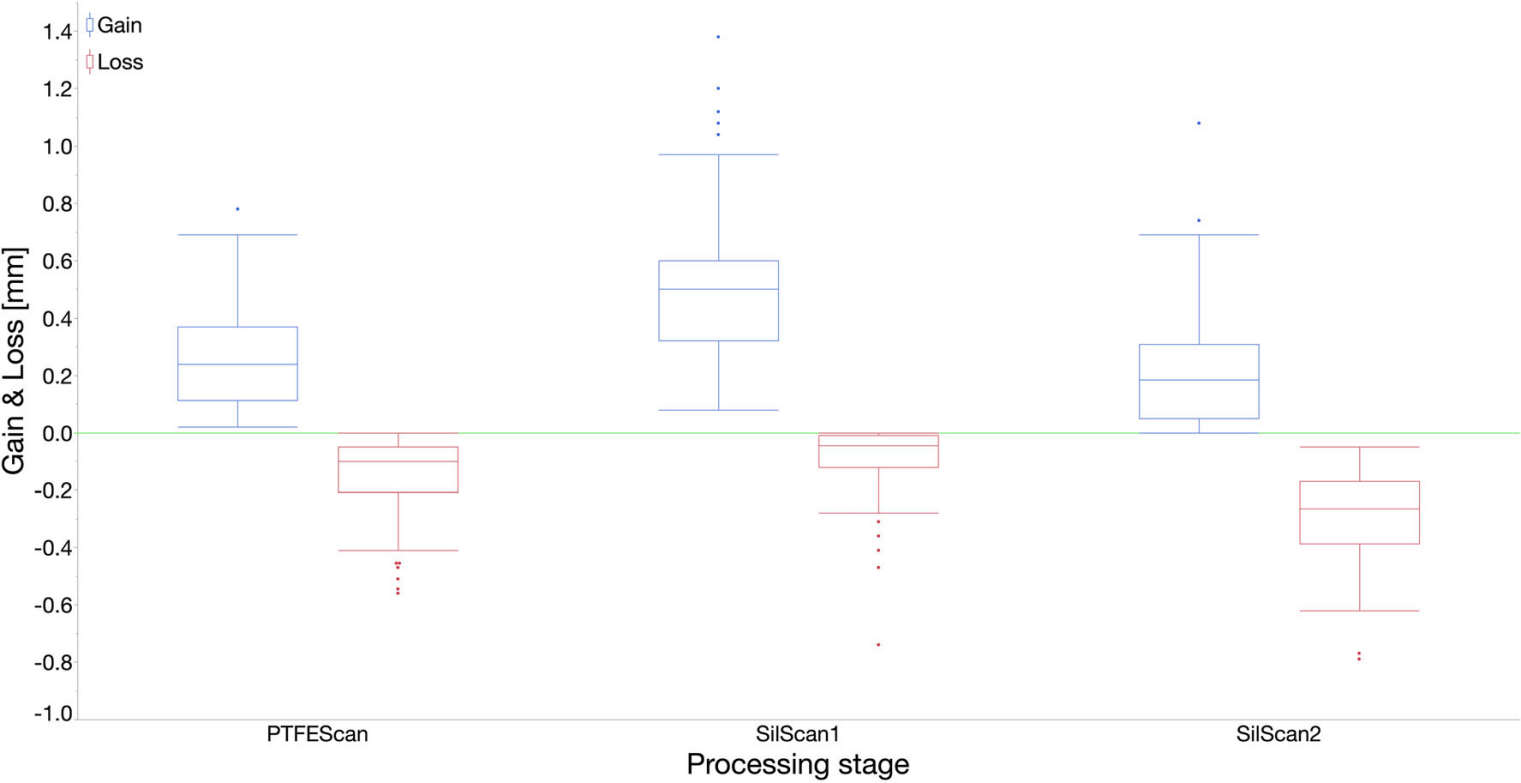
Boxplot showing the mean maximum gain and loss of the six evaluated section planes of each processing stage and stamp technique in comparison to the unimpaired occlusal surface

The mean maximum gain perpendicular to the occlusal plane in comparison to the unimpaired occlusal surface was (0.51 ± 0.03) mm for *SilScan1*, (0.26 ± 0.02) mm for *PTFEScan*, and (0.22 ± 0.02) mm for *SilScan2* (Fig. 3 and Fig. 6). The differences between *SilScan1* and both groups with restored class I cavities (*SilScan2* and *PTFEScan*) were significant with p < 0.0001. The difference between *SilScan2* and *PTFEScan* was not significant (p = 0.31).

The mean maximum loss perpendicular to the occlusal plane in comparison to the unimpaired occlusal surface was (−0.09 ± 0.01) mm for *SilScan1*, (−0.15 ± 0.02) mm for *PTFEScan*, and (−0.29 ± 0.02) mm for *SilScan2* (Fig. 3 and Fig. 6). All differences were significant (all p < 0.001).

## Discussion

This study is the first that acquired intraoral scanner data and evaluated them three-dimensionally to assess the quality of two stamp techniques in terms of restoring the original anatomy. The study by Rukmo et al. 35 years ago [15] is the only one that addressed this problem, but merely with the “analogous” methods of that time. In those days, silicone impressions of the occlusal surface were cut at exactly defined positions before (baseline) and after (follow-up) the placement of the restorations and the dimensional differences were measured under the microscope. The experimental effort that was necessary is still impressive today, and due to this, it can be easily understood that there has never been another attempt at this kind of study.

In contrast, the software *OraCheck 5.0.0* (Dentsply Sirona Deutschland GmbH, Bensheim, Germany) used in this study provides volume measurement [20]. The software superimposes two virtual optical scans (e.g., baseline and follow-up) using the best-fit method [21]. Since at least 80 % of the tooth surfaces in this study were not altered, the differences of superimposition are below 5 μm [22, 23]. Furthermore, scanner accuracy is above this value and depends upon the digitalized area and the scanner type. Ryakhovskiy et al. showed for a single tooth scan with the *CEREC Omnicam* (Dentsply Sirona Deutschland GmbH, Bensheim, Germany) a trueness of (25 ± 1) μm and a precision of (38 ± 3) μm [24], and Lee et al. demonstrated a trueness of (14 ± 1) μm and a precision of (13 ± 4) μm [25]. An explanation for the differences may be the software version, which is not mentioned in the paper of Ryakhovskiy et al. Indeed, Haddadi et al. showed that the software version may have an impact on the accuracy of the scan [26]. Therefore, the latest version available at the time, *CEREC SW 5.1.1* (Dentsply Sirona Deutschland GmbH, Bensheim, Germany), was used in the present study. Dettwiler et al. showed for measurements with *OraCheck* software that the measurement accuracy of the method is 31 ± 12 μm when the proportion of the overlap distance of 0.0–0.1 mm for the superimposition is greater than 95 % [27]. In this study, the proportion of the overlap distance of 0.0–0.1 mm was between 97 and 99 %. In summary, the experimental set-up has a high accuracy in comparison with the former approach and is suitable for the assessment of minor volumetric changes.

When using the silicone stamp technique, excess can be removed only after light curing. This was the reason why, in contrast to the PTFE stamp technique, a further surface scan was carried out at this stage of processing. As expected, the increase in volume (Fig. 5) in comparison to the unimpaired occlusal surface was greatest in the *SilScan1* group. An increase in volume was also measured in the *PTFEScan* group. However, this increase was only 23 % of the increase in the *SilScan1* group. The *SilScan2* group was the only one in which the volume decreased in comparison to the unimpaired occlusal surface. Nevertheless, it should be taken into account that the volume changes presented here are net volumes. For example, an RBC excess of 5 mm^3^ and a structure loss of 2 mm^3^ are quantified as a net volume change of 3 mm^3^. In this study, the areas with deficit or excess were (compared to others [28]) too small and too distributed to be selected with the region tool and measured separately (Fig. 3).

To overcome this, the greatest gain and loss were measured perpendicular to the occlusal plane in six sectional planes, three in oro-buccal and three in mesio-distal alignment (Fig. 4). After finalization, there was no difference between the two stamp techniques with respect to the mean maximum gain. With both methods, the mean maximum gain ranged between 0.20 and 0.28 mm and the excess was mainly located in the area of the central fissures (Fig. 3). In contrast, the mean maximum gain in the *SilScan1* group was almost twice as high and was predominantly located in the area of the marginal ridges. Rukmo et al. measured a mean maximum gain between 0.39 and 0.58 mm, however, after finalization of the restorations [15].

As already expected from the volume measurement, the *SilScan2* group showed with −0.29 mm, a mean maximum loss twice as high as the *PTFEScan* group with −0.15 mm. In the *PTFEScan* group, there were also substance losses in some areas, despite the increase in the net volume. In both groups, these were located at the cusp slopes and can therefore be attributed to the finalization with diamond burs and/or polishers. Interestingly, there was also a substance loss in the *SilScan1* group, although its mean maximum loss was the lowest with −0.09 mm. Here the losses occurred in the center of the occlusal surface. This is probably due to the fact that the silicone stamp, which is softer than the employed flowable light-curing resin paste used with the PTFE stamp technique, was not sufficiently dimensionally stable in the area above the cavity, despite the moderate pressure of 5 N applied in this study.

With the limitation that this is an in vitro study and the teeth lacked a functional antagonist, the two stamp techniques assessed do not differ with regard to the remaining excess after finalization. The excess in this study is approximately half as large as in the study by Rukmo et al. [15]. The location of the excess in the central fissures and its layer thickness corresponded visually to that of fissure sealings without being explicitly tested in this study. Nevertheless, the loss of natural tooth structure was significantly greater with the silicone stamp technique. Another disadvantage of this technique was the deficit of RBC due to the lower stiffness of the polysiloxane bite registration material as shown in this study and the fact that polysiloxanes interfere with the polymerization reaction as shown by Pitta Lopes et al. [19]. At least, these disadvantages could be avoided by the use of more rigid light-curable materials [9, 10] or thermoplastically deformable occlusal transfer devices [11–13] such as *Bite-pref*. The advantage of polysiloxanes is that they are more tolerant toward undercuts than more rigid materials and they are present in many dental practices. The latter was also the reason why polysiloxane was chosen in this study as transparent stamp material. Furthermore, a simple class I cavity model was chosen in this study in order to identify the differences between the techniques using a simple study model. This facilitated standardization of the cavities and ensured that sufficient unaltered tooth surface was available for baseline and follow-up superimposition. The pit and fissure area was chosen as the ideal area to compare the two techniques due to the anatomical complexity of this particular area of the tooth. Moreover, it represents one of the preferred areas to use these methods to achieve anatomical and functional restorations (without occlusal interferences) in a more reproducible and easy and less time-consuming way. Therefore, these techniques can be beneficial for the rehabilitation of complex cases, as demonstrated in a case report presented by Meller and Walker [29].

In this work, a small cavity in an area of complex anatomy (the pit and fissures area) was chosen to simplify and standardize the study method. However, these techniques are well applicable for the reconstruction of areas with much larger defects such as the cusps of premolars and generally the anatomy of anterior teeth [29]. In the present study, healthy tooth surface was used for the fabrication of the stamp. From a clinical point of view, these procedures are applicable in cases of hidden caries in which the tooth surface remains intact. Moreover, in the case of larger defects with loss of tooth structure on the surface, reconstruction options by means of a wax-up (if necessary even adjusted in an articulator) are also possible for the fabrication of stamps. The latter allows the realization of complex cases, with the advantage of previously adjusted results in form and functionality to achieve more predictable results in less time [29].

## Conclusion

The PTFE stamp technique performed better than the silicone stamp technique in this study; i.e., less healthy tooth structure was removed during the finishing procedure, and better results were achieved in reproducing the original shape of the tooth.
